# A preliminary survey of *Trichinella* spp. in pigs raised under controlled housing conditions in Colombia: 2014–2016

**DOI:** 10.1051/parasite/2018023

**Published:** 2018-04-10

**Authors:** Jenny J. Chaparro-Gutiérrez, Edoardo Pozio, María A. Gómez-Morales, Anderson López, Jaime Mejia, Corina Zambrano, Diego Piedrahita, David Villar

**Affiliations:** 1 CIBAV research group, Veterinary Medicine School, Faculty of Agrarian Sciences, University of Antioquia, Carrera 75 No 65-87, Medellín Colombia; 2 European Union Reference Laboratory for Parasites, Istituto Superiore di Sanità, 00161 Rome Italy; 3 Asociación Porkcolombia-FNP, Ceniporcino, Bogotá Colombia

**Keywords:** *Trichinella* spp, domestic pig, rat, Colombia, epidemiology, artificial digestion, serology

## Abstract

A preliminary survey of *Trichinella* spp. infection was conducted in Colombian swine herds between 2014 and 2016. A total of 1,773 pigs reared on farms under controlled housing conditions and processed in 34 slaughterhouses were tested either by the artificial digestion of pooled muscle samples (n = 1,173) or by serology (n = 600). In addition, 550 rats trapped on 29 swine farm premises were also tested by artificial digestion. No positive pig samples were detected. Similarly, no *Trichinella* spp. muscle larvae were detected in rats. These results are in agreement with the lack of historical *Trichinella* infection reports in domestic and wild animals and humans in Colombia. However, a more extensive epidemiological investigation and a continuous surveillance program are needed to continue declaring swine herds in Colombia free of *Trichinella* infection.

## Abstract (fr)

Une enquête préliminaire sur les infections dues à *Trichinella* spp. a été conduite chez des troupeaux de porcs colombiens entre 2014 et 2016. Un total de 1773 porcs élevés dans des fermes en condition de stabulation contrôlée et traités dans 34 abattoirs ont été testés par la méthode de digestion artificielle d’échantillons collectifs de muscles (n = 1173) ou par sérologie (n = 600). De plus, 550 rats piégés dans les locaux de 29 fermes d’élevage de porcs ont été testés aussi par digestion artificielle. Aucun échantillon de porc ne s’est révélé positif. Également, aucune larve musculaire de *Trichinella* n’a été détectée chez les rats. Ces résultats sont en accord avec l’absence historique de déclarations d’infections de *Trichinella* chez les animaux domestiques et sauvages et chez l’homme en Colombie. Cependant, il est nécessaire de conduire une enquête épidémiologique plus étendue et un programme de surveillance ininterrompu pour pouvoir continuer à déclarer les porcs colombiens libres de l’infection par *Trichinella*.

## Introduction

Parasites of the genus *Trichinella* show a cosmopolitan distribution on all the continents except Antarctica [21]. In South America, these zoonotic pathogens are endemic in Argentina and Chile, where they circulate among a large number of animals including pigs, which are the source of human trichinellosis outbreaks [13,17,20]. These nematodes were also documented in rats in Peru and Uruguay in the first half of the 20^th^ century [14]. In Bolivia, *Trichinella* spp. larvae have never been reported in animals or humans, but anti-*Trichinella* antibodies were detected in sera from domestic pigs in different regions of the country [19].

In Colombia, *Trichinella* spp. larvae were not detected in 800 pigs slaughtered in Bogotà in 1930 and anti-*Trichinella* antibodies were not detected in the sera of patients with eosinophilia in the 1960s [1]. According to Neghme and Schenone, trichinellosis was never documented in Colombia up to 1970 [14]. It follows that pigs reared in Colombia are considered to be free of *Trichinella* spp. even though there are no reports on epidemiological investigations in this domestic animal in recent decades, and no surveillance for the presence of *Trichinella* has been implemented in Colombian abattoirs. Thus, the absence of official and/or published reports might not reflect the real situation, warranting the conduct of epidemiological surveys to demonstrate the “*Trichinella*-free” status of Colombian swine herds.

Many wild and synanthropic animals can harbor *Trichinella* spp. and serve as a potential source of infection to domestic animals. Among others, rodents of the genus *Rattus* (*e.g.*, *Rattus norvegicus*) are considered a vector of *Trichinella spiralis* in the domestic habitat, favoring the transmission among backyard-raised pigs [10,20]. At the worldwide level, domestic pigs are the main source of *Trichinella* spp. infection for humans, followed by wild swine [13].

The Colombian swine population is about 4.5 million animals, of which about four million are slaughtered annually [18]. Recent directives for exporting pig carcasses to international markets have prompted the implementation of mandatory testing at national swine abattoirs using the internationally approved method of artificial digestion of pooled samples [2,3,16,24]. In addition, to recognize Colombia as free from *Trichinella* spp. in domestic swine, an effective reporting system and surveillance program must be implemented to confirm the absence of these pathogens [15]. The aim of the present study was to investigate the presence of *Trichinella* spp. in domestic pigs slaughtered in the major swine-producing areas of Colombia.

## Materials and Methods

### Study design and sampling

**Figure 1:**
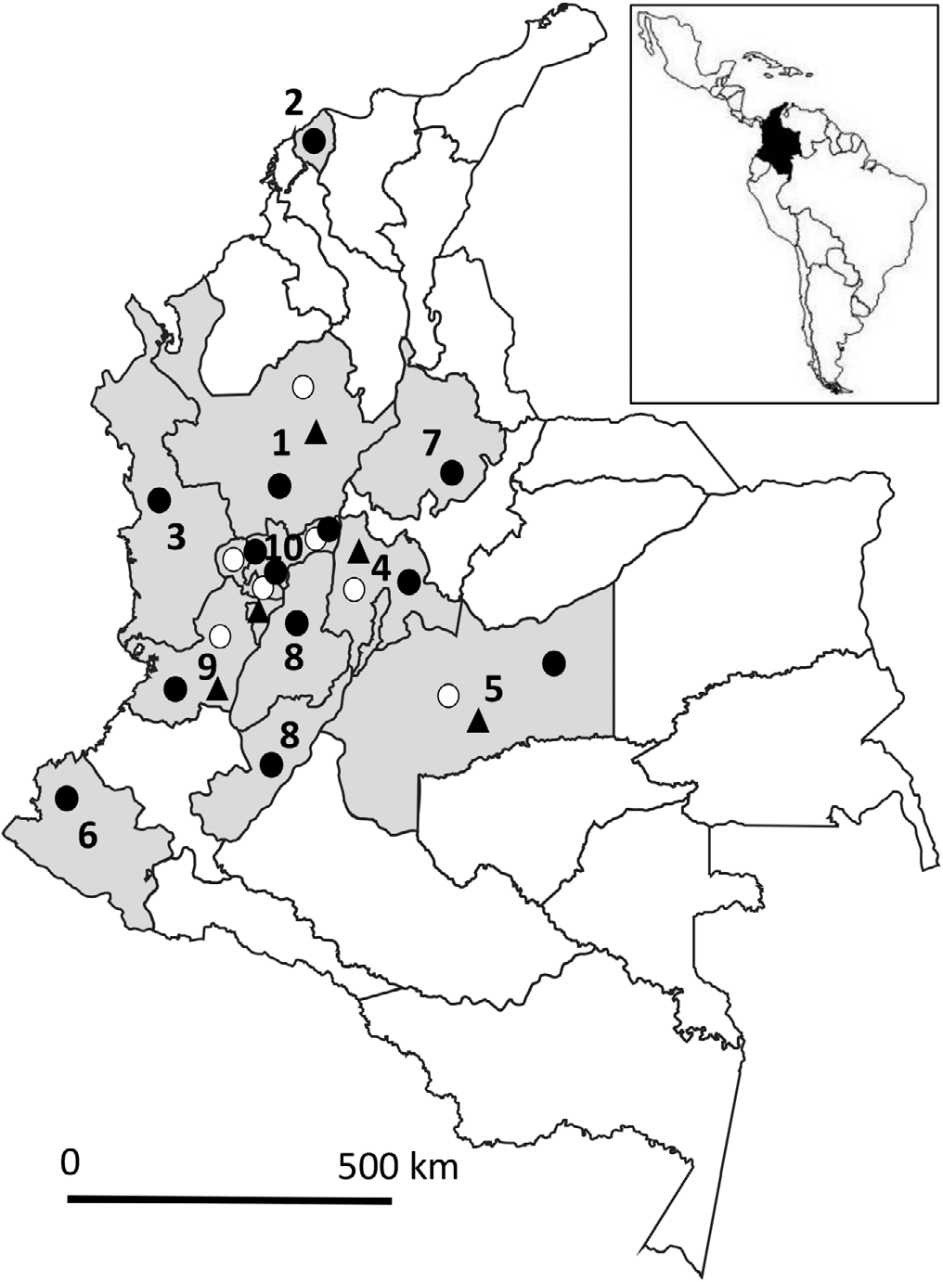
Map of Colombia showing the slaughterhouses where muscle (black circles) and serum samples (open circles) were collected from slaughtered pigs, and pig farms (black triangles) where synanthropic rats were trapped from 2014 to 2016. Colombian states: 1, Antioquia; 2, Atlántico; 3, Choco; 4, Cundinamarca + DC; 5, Meta; 6, Nariño; 7, Santander; 8, Tolima Y Huila; 9, Valle del Cauca; and 10, Zona Cafetera.

The survey was conducted in 34 main swine slaughterhouses located in 10 of 32 states across Colombia and processing 80% of the national pig production (Fig. 1). Complete data are not available on the farms of origin, since dealers deliver pigs from several farms to slaughterhouses. A total of 1,173 porcine muscle samples and 600 porcine serum samples were tested by the artificial digestion assay and ELISA, respectively. Muscle and serum samples were collected from different pigs. Most tested animals were offspring of the PIC Camborough^®^ sow breed and PIC 337-410 boar breed, raised under controlled housing conditions, *i.e.* in a confinement system and fed by controlled feed, according to the International Commission on Trichinellosis and OIE Guidelines [11,16]. Considering that *Trichinella* spp. has never been documented in Colombian pigs, the prevalence was expected to be negligible, *i.e.* lower than one per million. Since the total population is about 4.5 million and about 4 million are slaughtered per year, the number of animals that should be tested to confirm a “negligible risk match” would be the total number of slaughtered animals. Due to cost limitations and the presence in the country of only one laboratory accredited by the Colombian regulatory authority “INVIMA” (counterpart of the American USDA) to perform the artificial digestion assay and the serological test, the number of pigs that were sampled at every slaughterhouse was chosen based on the number of pigs processed by each plant on an annual basis. Sampling was conveniently divided into three groups according to the number of pigs processed per plant. Therefore, for plants slaughtering 3,000–9,999, 10,000–100,000 and >100,000 pigs/year, 150, 252 and 760 pigs were sampled, respectively. For the first group (n = 150), 7–10 pigs were sampled starting from every 35^th^ slaughtered animal. Similarly, 16–17 and 76–80 pigs were sampled from the second (n = 252) and third (n = 760) groups, each time starting at the 221 and 271 slaughtered pig.

At the end of sampling, a total of 1,173 pigs were sampled, *i.e.* 150 + 252 + 760 as previously established, plus 11 additional pigs.

Muscle tissues (diaphragm pillars and masseters) were stored at + 4 °C for no more than three days until digestion. Pigs tested by either methodology were different, although the selection criteria determining which animals to sample were similar, as described above.

Serum samples (5 mL per animal) were collected from pigs reared under controlled housing conditions and slaughtered in five Colombian states (Antioquia, 1; Cundinamarca, 1; Valle del Cauca, 1; Zona Cafetera 3; and Meta, 1), following the same sampling criteria reported above for the muscle collection. The number of serum samples to be collected was determined to be 600 on the basis of the available ELISA kits. As part of a study on *Toxoplasma gondii* and *Leptospira* spp. (Chaparro-Gutiérrez J.J *et al.*, unpublished data), pooled muscle samples (10–20 g from the whole carcass of 5–10 rats) of 550 rats (74% *Rattus norvegicus* and 26% *R. rattus*) trapped within the premises of 66 swine farms in seven Colombian states, were also tested for *Trichinella* spp. infection by the artificial digestion assay.

### Artificial digestion assay

The artificial digestion assay for the detection of *Trichinella* spp. larvae in muscle samples was performed according to Commission Implementing Regulation (EU) 2015/1375, and was validated in the laboratory of the Veterinary Medicine School, Faculty of Agrarian Sciences, University of Antioquia, Medellín [3]. The sensitivity of the assay using 5 g of muscle tissue per pig is 1 larva per gram of tissue [6]. Briefly, a 5-g sample of tongue and a 5-g sample of diaphragm pillars from each carcass of 10 pigs were blended in a pool of 100 g. Samples were digested in 2 L of 47 °C tap water with 0.2% hydrochloric acid and 0.5% pepsin (1:10,000 NF, PanReac AppliChem). The digest was stirred for 30 min at 44-46 °C in a 3-liter glass beaker on a magnetic stirrer plate. After artificial digestion, the solution was poured through a sieve (mesh size 180 μm) into a separatory funnel. After 30 min of sedimentation, 40 mL were collected into a 50 mL Falcon tube and allowed to sediment for 10 minutes. The supernatant (30 mL) was discharged and the remaining 10 mL were examined on a gridded Petri dish. The Falcon tube was further rinsed with tap water, which was added to the Petri dish. The sediment was examined at 40 X magnification for the presence of *Trichinella* spp. larvae by a stereomicroscope.

### Serology

Serum samples were tested to detect IgG against *Trichinella* spp. using a commercial ELISA kit based on the excretory-secretory (E/S) antigens (pig type *Trichinella* Ab; Qiagen, Hilden, Germany). The assay was performed in a three-step protocol as follows: 1) each serum was diluted 1:100 and incubated at room temperature for 60 min on plates with E/S antigens of *T. spiralis*; 2) after three buffer washes, a peroxidase-labelled anti-pig IgG was used as secondary antibody and incubated for 30 min; 3) after three buffer washes, a chromogen substrate was added and the optical density of the samples was measured at 450 nm wavelength. The results were calculated with reference to the positive and negative controls, with an S/P ratio in optical density exceeding 0.30 considered as positive.

## Results and Discussion

The number of tested pigs by Colombian state and year is shown in Table 1. No infection by *Trichinella* spp. larvae was detected in any of the 1,773 pigs tested by either the artificial digestion assay (n = 1,173) or by ELISA (n = 600). Similarly, muscle samples of the 550 rats tested negative for *Trichinella* ssp. larvae. This is consistent with the expected prevalence (less than 1 infected pig per million slaughtered pigs). However, it is of note that with the number of tested pigs (n = 1,773) established *a priori* on the basis of funding resources, the upper 95% confidence interval of the infected pigs in the population is around 2/1,000 pigs, corresponding to around 9,000 infected pigs of the 4.5 million in the country.

**Table 1:** Pigs tested for *Trichinella* infection by the artificial digestion assay or by ELISA per Colombian states in 2014-2016.

State	N. of tested pigs (N. of slaughterhouses)	
	
	ELISA	Digestion assay
Antioquia	374 (16)	648 (15)
Cundinamarca	96 (8)	181 (4)
Valle del Cauca	75 (6)	176 (3)
Zona Cafetera	45 (3)	48 (3)
Meta	10 (1)	30 (2)
Atlántico		32 (2)
Chocó		16 (1)
Santander		8 (1)
Tolima y Huila		18 (2)
Nariño		16 (1)
Total	600 (34)	1173 (34)

However, this is the first survey conducted across the major swine producing areas of Colombia. Colombian regulations do not require any mandatory *Trichinella* spp. surveillance, but this may soon change with new international trade agreements. Considering the cost of routine carcass examination for *Trichinella* spp. by the artificial digestion assay, and that no positive samples were detected so far in Colombian pigs raised under controlled housing conditions, serological testing could be suitable for *Trichinella* spp. surveillance in pigs, according to the International Commission on Trichinellosis [7]. Since serology has been shown to occasionally yield false-positive results, or sometimes positive results with very low to undetectable levels of larval burden [4,23], it would be warranted that any seropositive pig be further confirmed by the artificial digestion assay. A study to evaluate the reproducibility and validation of an ELISA has shown that, when the recommended protocol is strictly followed, a negative result is an excellent indicator of the absence of infection, with a specificity of 98.29% [8]. Since anti-*Trichinella* IgG are detectable in both serum samples and meat juice samples (tested at 1/10 dilution), meat juice could be used as an alternative sample matrix for serological screening [9,12].

However, considering the endemic situation in other South American countries such as Argentina and Chile [20,22], the effects of globalization, and the possible presence of a sylvatic cycle in Colombia, which can be the source of infection of pigs raised in backyards or free-ranging pigs, a continuous surveillance program is warranted to continue declaring swine herds raised under controlled housing conditions in Colombia free of *Trichinella* spp. infection. In the last 20 years in the European Union, *Trichinella* spp. infection has been detected only in backyard or free-ranging pigs and never in pigs raised under controlled housing conditions, regardless of the prevalence in wild animals, which can be high [5]. A similar epidemiological pattern of *Trichinella* spp. infections in domestic pigs has also been observed in other countries around the world [20].

## Conflict of interest

The authors declare that they have no conflicts of interest in relation to this article.

## References

[1] Botero D, Salazar O. 1964 Triquinosis en Colombia. Antioquia Medica, 14, 723-726.

[2] CONPES 3558. 2007. Departamento nacional de Planeación: Política Nacional de Sanidad e Inocuidad para la cadena porcícola. (www.dnp.gov.co).

[3] European Commission. 2015. Commission implementing regulation 2015/1375 of 10 August 2015 laying down specific rules on official controls for *Trichinella* in meat. Official Journal of the European Commission Legislation, 212, 7-34.

[4] Chávez-Larrea MA, Dorny P, Moeller L, Benítez-Ortiza W, Barrionuevo-Samaniego M, Rodríguez-Hidalgo R, Ron-Romána J, Proaño-Péreza F, Victor B, Brandt J, Kapel C, Borchgrave J. 2005 Survey of porcine trichinellosis in Ecuador. Veterinary Parasitology, 132, 151-154. 15978724 10.1016/j.vetpar.2005.05.045

[5] EFSA. 2016. The European Union summary report on trends and sources of zoonoses, zoonotic agents and food-borne outbreaks in 2015. EFSA Journal, 14, 4634. 10.2903/j.efsa.2017.5077 PMC7009962 32625371

[6] Forbes LB, Gajadhar AA. 1999 A validated *Trichinella* digestion assay and an associated sampling and quality assurance system for use in testing pork and horse meat. Journal of Food Protection, 62, 1308-1313. 10571321 10.4315/0362-028x-62.11.1308

[7] Gamble HRE, Pozio F, Bruschi K, Nöckler C, Gajadhar AA. 2004 International Commission on Trichinellosis: recommendations on the use of serological tests for the detection of *Trichinella* infection in animals and man. Parasite, 11, 3-13. 15071823 10.1051/parasite/20041113

[8] Gómez-Morales MA, Ludovisi A, Pezzotti P, Amati M, Cherchi S, Lalle M, Pecoraro F, Pozio E. 2009 International ring trial to detect anti-*Trichinella* IgG by ELISA on pig sera. Veterinary Parasitology, 166, 241-248. 19819075 10.1016/j.vetpar.2009.09.005

[9] Gómez-Morales MA, Ludovisi A, Amati M, Bandino E, Capelli G, Corrias F, Gelmini L, Nardi A, Sacchi C, Cherchi S, Lalle M, Pozio E. 2014 Indirect versus direct detection methods of *Trichinella* spp. infection in wild boar (*Sus scrofa*). Parasites & Vectors, 7, 171. 24708795 10.1186/1756-3305-7-171 PMC3995759

[10] Gottstein B, Pozio E, Nöckler K. 2009 Epidemiology, diagnosis, treatment, and control of trichenollosis. Clinical Microbiology Reviews, 22, 127-145. 19136437 10.1128/CMR.00026-08 PMC2620635

[11] International Commission on Trichinellosis. Recommendations for Pre-Harvest Control of *Trichinella* in food animals. http://www.trichinellosis.org/Guidelines.html.

[12] Møller LN, Petersen E, Gamble HR, Kapel CMO. 2005 Comparison of two antigens for demonstration of *Trichinella* spp. antibodies in blood and muscle fluid of foxes, pigs and wild boars. Veterinary Parasitology, 132, 81-84. 15982820 10.1016/j.vetpar.2005.05.032

[13] Murrell KD, Pozio E. 2011 Worldwide occurrence and impact of human trichinellosis, 1986-2009. Emerging Infectious Diseases, 17, 2194-2202. 22172230 10.3201/eid1712.110896 PMC3311199

[14] Neghme A, Schenone H. 1970. Trichinosis in Latin America, in Trichinosis in Man and Animals, Gould SE Editor. C.C. Thomas: 1; Springfield, Illinois. p. 407-422.

[15] Nöckler K, Kapel CMO. 2007. Detection and surveillance for *Trichinella*: meat inspection and hygiene, and legislation in FAO/WHO/OIE guidelines for the surveillance, management, prevention and control of trichinellosis, Dupouy-Camet J, Murrell KD, Editors. World Organization for Animal Health Press: Paris, France. p. 69-97.

[16] OIE. 2013. Chapter 8.14, Infection with *Trichinella* spp., in Terrestrial Animal Health Code. World Organization for Animal Health: Paris, France. p. 1-4.

[17] Ortega-Pierres MD, Arriaga C, Yépez-Mulia L. 2000 Epidemiology of trichinellosis in Mexico Central and South America. Veterinary Parasitology, 93, 210-225. 10.1016/s0304-4017(00)00342-3 11099838

[18] Porkcolombia-FNP, 2016. Boletin económico: Análisis de coyuntura del sector porcicultor del año 2016 y perspectivas del 2017. https://asociados.porkcolombia.co/porcicultores/images/porcicultores/informes/2016/Inf_Economico_2016.pdf. Retrieved 28/11/2017.

[19] Pozio E. 2007 World distribution of *Trichinella* spp. infections in animals and humans. Veterinary Parasitology, 149, 3-21. 17689195 10.1016/j.vetpar.2007.07.002

[20] Pozio E. 2014 Searching for *Trichinella*: not all pigs are created equal. Trends in Parasitology, 30, 4-11. 24314577 10.1016/j.pt.2013.11.001

[21] Pozio E, Zarlenga DS. 2013 New pieces of the *Trichinella* puzzle. International Journal for Parasitology, 43, 983-997. 23816802 10.1016/j.ijpara.2013.05.010

[22] Ribicich M, Gamble HR, Bolpe J, Sommerfelt I, Cardillo N, Scialfa E, Gimenez R, Pasqualetti M, Pascual G, Franco A, Rosa A. 2009 Evaluation of the risk of transmission of *Trichinella* in pork production systems in Argentina. Veterinary Parasitology, 159, 350-353. 19041182 10.1016/j.vetpar.2008.10.072

[23] Roesel K, Nöckler K, Baumann MPO, Fries R, Dione MM, Clausen PH, Grace D. 2016 First report of the occurrence of *Trichinella*-specific antibodies in domestic pigs in central and eastern Uganda. PLoS One, 11, e0166258 27870858 10.1371/journal.pone.0166258 PMC5117603

[24] Rossi P, de Smet K, Pozio E. 2017 Detection of *Trichinella* larvae in meat: comparison of ISO 18743:2015 with regulation (EU) 2015/1375. Food Analytical Methods, 10, 634-639.

